# Medical News and Items

**Published:** 1875-10

**Authors:** 


### Medical News and Items.

A Daniel Come to Judgment!—The N. Y. *Medical Record* of August 7th, in a critique upon Dr. Johann Steiner's Compendium of Children's Diseases, translated into English by Mr. Lawson Tait, perpetrates some inexcusable blunders.

The author of the critical (?) review to which we refer, objects to the phrase "exhibition of a drug," and decides that "administration" is the proper term to be employed in such a connection. If Dr. Noah Webster be considered an authority in such matters, Mr. Tait is quite correct in his use of the language. Here is the definition given in the Dictionary *verbatim:*

"3. (Med.) To administer as a remedy; as, to *exhibit* calomel."

Our critic further considers "contraction" an imperect translation, when "contracture" should be employed. There is no such word as "contracture" in proper English medical phraseology.

The same writer would substitute for "exaggerated respirations," "accelerated"—a substitution which would entirely change the meaning of the author.

He would also change "Canton Schweiz" and "Schweiz" into "Switzerland." Now, "Canton Schweiz" no more refers to "Switzerland" than does "Borriboola Gha"! We recommend our critic to the study of geography.

Here is a sample of the mother tongue of the same reviewer: "Children predisposed to scrofula entirely on animal food," "by no means so full as the American reader would like to see," etc.

This is hypercriticism, but it is the sole remedy for hypercritics.

Distinction and Difference.—The Cincinnati *Clinic* of Sept. 11, comments as follows upon the consolidation of the two medical journals of this city: "So far as we can judge from the present number, the Journal is now an organ of the Press Association, instead of a Medical College: to an outsider this is a distinction without a difference."

The Chicago Medical Press Association, of which this journal is the official organ, represents the entire medical profession of Chicago in its corporators and stockholders. Instead, therefore, of being the organ of a single medical college, the Journal and Examiner is to-day the organ and representative, not only of all the regular medical colleges, but also of the other medical institutions of Chicago. This would seem to be a distinction *with* a difference.

An illustration of the failure of *both* distinction and difference may be found in the leading article of this same number of the *Clinic*, where, out of some sixteen published formulæ, at least ten are expressed in a singular admixture of English and Latin words. Some of the latter, too, are in a bad case. "To an outsider" the *Clinic* would appear to make neither distinction nor difference between the two languages. We commend our sprightly cotemporary to the Report on Medical Literature in the Transactions of the American Medical Association for 1873.

The new County Hospital—or the two pavillions thereof now being erected—is progressing favorably. The walls of both buildings are now nearly ready to be roofed. How long it will be before the wards will be ready to receive patients, nobody knows, but we are informed that architect Cochrane promises the Board of Commissioners that the building shall be completed by mid-winter. Of course after the ward-rooms are finished, several weeks must elapse before they will be sufficiently dry to make it safe to fill them with sick people.

The Board of Commissioners of Cook County have done a very sensible thing to loan the city sufficient funds to enable the latter to thoroughly sewer and fill up to grade the streets bounding on the north and east of the new hospital lot. The Board have moved in the matter of a new hospital very slowly, but many of their late actions in the premises have been marked with commendable wisdom.

In the Mount Sinai Hospital, New York City, a new treatment for articular rheumatism is said to have been adopted, which consists "in packing the patients with blankets wrung out of hot water and changed as often as their temperature falls." The results are said to be as good as those obtained from the use of cold water and ice.—*N. Y. Med. Jour.*, Sept., 1875.

The new building for Rush Medical College will be commenced in a few weeks. The plans are now nearly complete. The building will be 68 by 82 feet on the ground; will reach nearly 80 feet toward the clouds, and will have four stories above a nine-foot basement. The style of architecture is, in a general way, Gothic. The building will be surmounted by a mansard roof, furnished with large dormer windows to give light to the upper lecture room. Each of the two lecture rooms will have capacity for seating 450 people. The new college lot is on the corner of Wood and Harrison streets, diagonally opposite the new County Hospital. We learn that it is intended to have the building completed by the time the County Hospital is ready for occupancy.

The *Chicago Journal of Nervous and Mental Diseases* is at present our only cotemporary, representative of regular medicine in this city. The reputation which it has already established in this country and abroad, is based not only upon the intrinsic excellence of its original researches, but upon its value as a mirror of neurological science. We are as justly proud of its success as its editors might well be; and take pleasure in commending its pages to our friends and subscribers.

The Board of Directors of the Central Free Dispensary of West Chicago have contracted to establish in the building of Rush College, as soon as completed, a free dispensary, to be a permanent institution. This arrangement is made to carry out the provisions of the will of the late Jno. Phillips, who bequeathed a considerable fund in trust to the trustees of Rush College, for dispensary purposes for the sick poor of Chicago. Almost the entire first floor of the College is to be set aside for the use of the dispensary. The income from the bequest referred to is to be devoted to the maintenance of this dispensary.

An exceedingly interesting paper on the existence of Motor Centres in the Cortical Substance of the Brain was read by the President of the American Neurological Association before the Chicago Society of Physicians and Surgeons on the evening of September 13th. It was received with marked attention by the unusually large number of medical gentlemen who had assembled to hear it. We shall present a brief abstract of the same in the Society Report in our next issue; and desire to state that the paper will be published *in extenso* in the October number of the *Chicago Journal of Nervous and Mental Diseases.*

Death of Sir Charles Locock.—The *London Echo* contains the announcement of the death of Sir Charles Locock, Baronet, the first physician accoucheur to Queen Victoria. He was born in 1799, and graduated in Edinburg in 1821. After going to London, he was selected, above all others, at the advice of Sir James Clarke, as physician-accoucheur. He was also a Fellow of the College of Physicians, at Edinburg, President of the Royal Medical and Chirurgical Society in 1857, was appointed Honorary President of the Obstetrical Society in 1863, and in 1864 was elected a Fellow of the Royal Society. He attended the Queen at the birth of every one of her nine children.

The Vienna Faculty.—The council of Medical Professors at Vienna, at its last sitting, appointed Dr. Mayerhofer Extraordinary Professor of Gynecology, and Dr. Rosenthal of Neuropathology and Electro-Therapeutics. Dr. Wertheim will probably also be appointed an Extraordinary Professor of Dermatology and Syphilis. Drs. Kaposi, Auspitz, and Neumann, formerly *privat docenten*, have been appointed Extraordinary Professors of Dermatology and Syphilis. Dr. Carl Stoerk, *privat docent*, has been appointed Extraordinary Professor of Laryngoscopy.—*Times and Gazette*, July 24, 1875.

A New Medical Term.—"In an article on the correspondence of the celebrated socialist, Proudhon, Dr. Pellarin, in quoting the passage—'a while ago I thought myself convalescent, I should rather say *dévalescent*, for I am tending not towards health but towards disease,'—says that his readers (*Union Méd.*, June 10,) ought to feel obliged to him for noting this creation of so expressive a word by Proudhon."—*Union Médicale.*

The *N. Y. Med. Journal* for September, scores mercilessly the *N. Y. Med. Register* for 1875-6, for a number of defects and shortcomings, chief among which are the following: It is confused in its arrangement; it has no index; the advertisements interspersed through the book give it the appearance of a cheap business directory, and the typography is wretched.

The Pruritus of Variola.—Dr. Noel Guéneau de Mussey recommends the following ointment to allay the pruritus which accompanies the eruption of variola, and to prevent the patient from lacerating the skin by scratching: Bromide of Potassium, 3 grammes (45 grs.); Camphor, 30 centigrammes (4.5 grains); Cerate, 30 grammes (450 grains). To be applied daily.—*Union Médicale*, July 24; *Times and Gazette*, July 31, 1875.

By a rule recently adopted by the Trustees of the Hospital for Women and Children, hereafter *Internes* in that institution (women always) are to be appointed by competitive examination. This is as it should be.

Dr. F. C. Hotz has resigned his position as Oculist and Aurist to the Cook County Hospital. Dr. W. T. Montgomery succeeds temporarily to the service.

Dr. Wm. C. Lyman, who left town a few weeks ago afflicted, as it was feared, with a fatal malady, has returned and resumed his practice. He is as hearty, to all appearance, as he ever was.

